# The wrist/palm forearm ratio is less reliable in diabetic patients with carpal tunnel syndrome than in non-diabetic patients

**DOI:** 10.1016/j.cnp.2026.09.005

**Published:** 2026-09-26

**Authors:** Marianna Tóth, Zsuzsanna Arányi

**Affiliations:** a Dept. of Neurology, Semmelweis University, Budapest, Hungary; b Dept. of Neurology, Vaszary Kolos Hospital, Petőfi u. 26-28, Esztergom 2500, Hungary

**Keywords:** Diabetes, Carpal tunnel, Nerve ultrasound, Median nerve, Cross sectional area, Wrist/palm forearm ratio

## Abstract

**Objective:**

This prospective study aimed to compare ultrasonographic parameters in diabetic and non-diabetic carpal tunnel syndrome and to analyze whether the findings are related to the presence of associated polyneuropathy.

**Methods:**

The cross-sectional areas (CSAs) of the median nerve were measured at three sites using high-resolution ultrasound in 63 patients with diabetes mellitus and carpal tunnel syndrome (73 hands), 146 non-diabetic patients with carpal tunnel syndrome (163 hands), and 50 controls (50 hands). The diabetic group included 13 patients with type 1 (15 hands) and 50 with type 2 diabetes (58 hands); of these, 43 also had polyneuropathy. Electrophysiological testing was performed to confirm carpal tunnel syndrome, determine its severity, and assess for polyneuropathy.

**Results:**

Wrist/palm cross-sectional areas (CSA-I, CSA-O) did not differ between the diabetic and non-diabetic groups. Wrist/palm–forearm ratio (WFR/PFR) was lower (*p* = 0.007), while forearm CSA was larger (*p* = 0.002) in diabetic patients compared to non-diabetic individuals, particularly in patients with type 2 diabetes and moderately severe carpal tunnel syndrome. WFR was lower in the diabetic subgroup with polyneuropathy than in the subgroup without polyneuropathy. Sensitivity, positive predictive value, and accuracy of the WFR/PFR were lower in diabetic than non-diabetic patients; however, these differences decreased when CSA-I/CSA-O values were combined with WFR/PFR.

**Conclusions:**

The wrist/palm–forearm ratio is less reliable for diagnosing carpal tunnel syndrome in diabetic than non-diabetic patients, particularly in moderately severe carpal tunnel syndrome and type 2 diabetes.

**Significance:**

It is to consider multiple parameters when performing ultrasound-based diagnosis of diabetes-related compressive neuropathies.

## Highlights

• Nerve cross-sectional area is a precise marker of carpal tunnel syndrome in diabetes.
• Wrist/palm-to-forearm ratio is a less accurate marker in diabetic patients.
• Combined application of ultrasound parameters enhances diagnostic accuracy.

## 1 Introduction

Nervous system reacts sensitively to metabolic changes due to its high energy demand *(*Edwards et al., 2008*)*. In addition, peripheral nerves damaged by metabolic disturbances are more susceptible to compression *(*Thomsen et al., 2009*).* This explains why diabetes is a known risk factor for compression neuropathies such as carpal tunnel syndrome (Pourmemari and Shiri, 2016*)*.

In early stages of nerve injury, functional abnormalities develop that can be detected by electrophysiological techniques, while structural changes occurring later can be examined using high-resolution ultrasound *(*Wong et al., 2004*)* Fig. 1, Fig. 2*.* In recent years, neurosonography has become an important complementary diagnostic tool alongside electrophysiology. It has been established that the cross-sectional area (CSA) of the median nerve at the carpal tunnel inlet is an accurate diagnostic parameter (Level A evidence) for carpal tunnel syndrome *(*Cartwright et al., 2012*;* Tai et al., 2012*)*. The wrist-to-forearm ratio (WFR), proposed by *Hobson-Webb (2008),* in which the patient serves as their own control, is another useful diagnostic parameter. According to observations of Paliwal et al. (2014)*,* when measurements taken at the tunnel outlet and the corresponding palm-to-forearm ratio (PFR) were analyzed together, diagnostic sensitivity increased by 19%. It was also noted that the median nerve tends to be more swollen at the outlet than at the inlet or within the tunnel *(*Csillik et al., 2016); therefore, including CSA values measured at the outlet can further improve diagnostic accuracy.

**Fig. 1:**
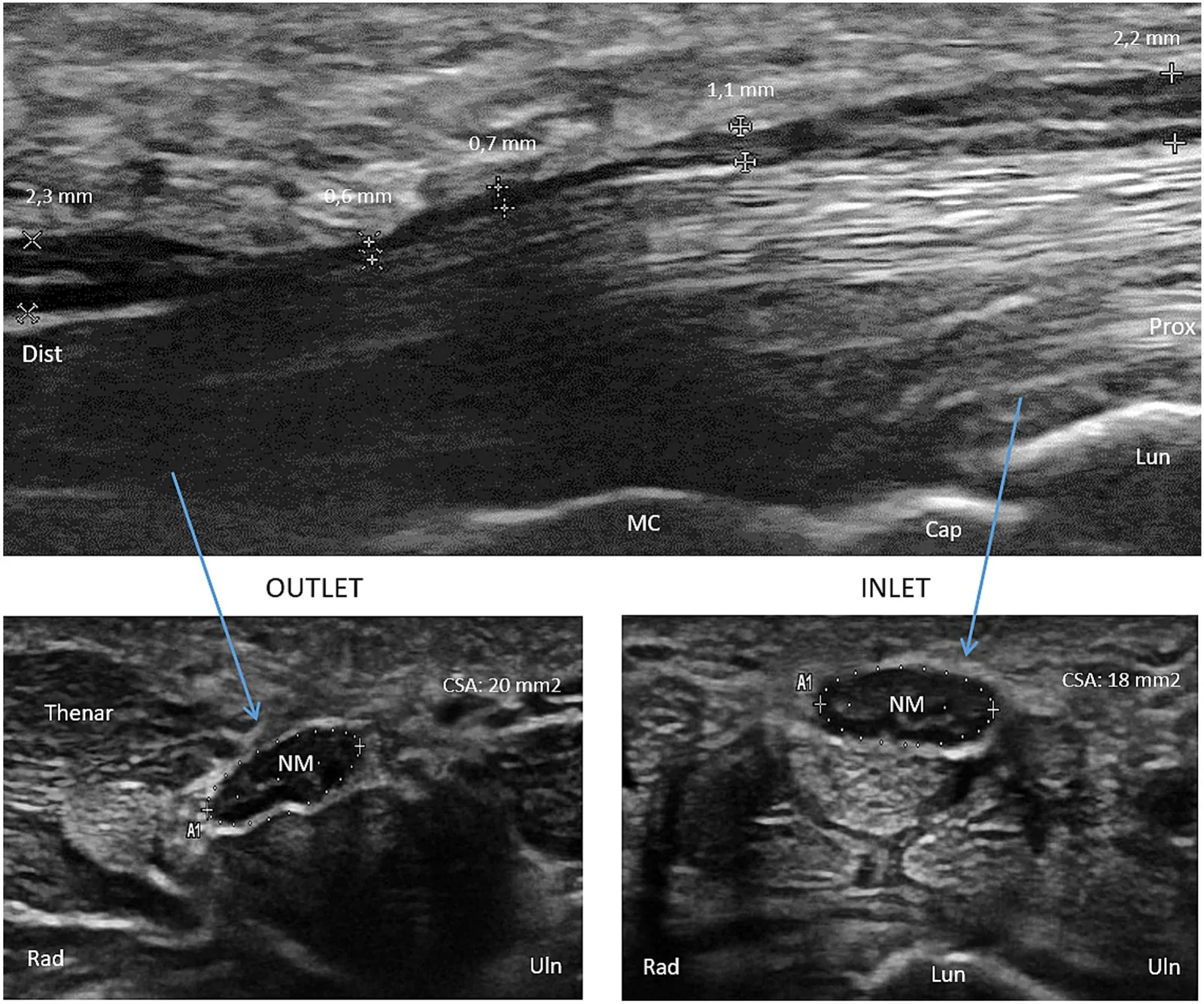
Cross-sectional ultrasound images of the median nerve at the carpal tunnel inlet (top) and at the forearm (bottom). In the patient with type 2 diabetes (right), the median nerve is mildly enlarged at the forearm with slightly inhomogeneous echotexture and swollen fascicles. In the patient with carpal tunnel syndrome without diabetes (left), the nerve at the forearm shows normal size and structure. At the carpal tunnel inlet, the median nerve in both patients is enlarged and hypoechogenic. Prox: proximal; Dist: distal; Rad: radial; Uln: ulnar; Lun: lunate bone; FDS: flexor digitorum superficial muscle.

**Fig. 2:**
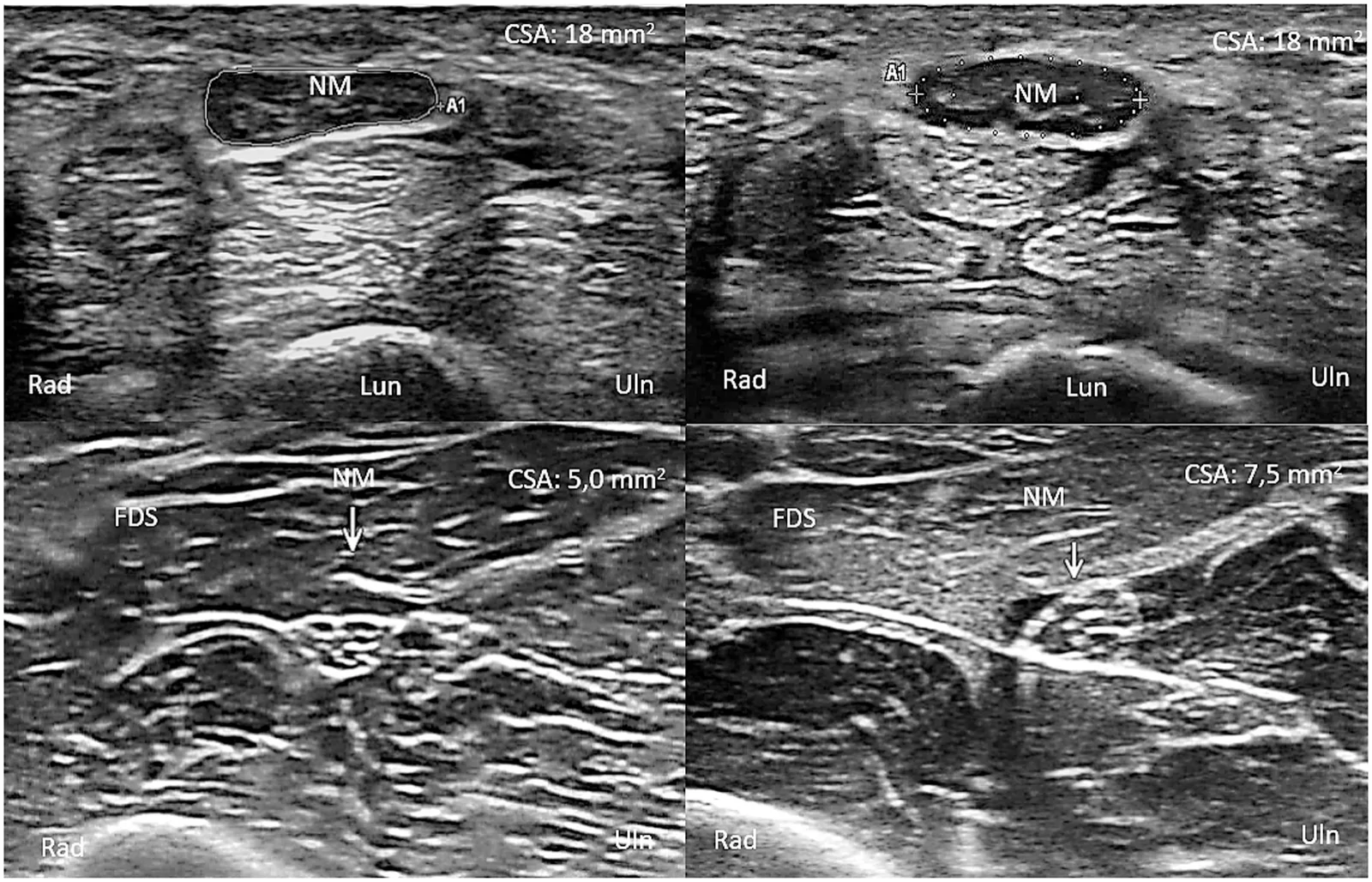
Ultrasound image of severe carpal tunnel syndrome. The longitudinal image (top) shows that the median nerve flattened along the entire length of the tunnel. In the cross-sectional images (bottom), the nerve has abnormal structure and enlarged at the inlet (right) and outlet (left). Rad: radial; Uln: ulnar; NM: median nerve; Lun: lunate bone; Cap: capitate bone; MC: metacarpus.

Neurosonography has demonstrated diabetes-related morphological abnormalities of peripheral nerves, the most commonly described features of which are enlarged cross-section and increased hypoechoic fraction (Breiner et al., 2017*;* Watanabe et al., 2010*)*. Some studies on carpal tunnel syndrome have reported increased wrist CSA in diabetic patients compared with non-diabetic individuals *(*Moon et al., 2014*;* Kotb et al., 2018*)*, while others have found no significant differences between the two groups *(*Kim et al., 2014*;* Tsai et al., 2013*)*. In their meta-analysis of the extensive literature, Chen et al. (2020) found no significantly greater nerve enlargement in diabetes-related carpal tunnel syndrome than in non-diabetic carpal tunnel syndrome.

In patients with diabetes examined for carpal tunnel syndrome, we occasionally observed a larger-than-expected median nerve CSA at the wrist or forearm. This raised the question of whether ultrasound is equally useful for diagnosing carpal tunnel syndrome in diabetic patients. Therefore, the aim of this prospective study was to evaluate the diagnostic accuracy of ultrasound parameters in diabetes-related carpal tunnel syndrome and to analyze whether the results are influenced by other factors such as the severity of tunnel syndrome or the presence of polyneuropathy.

## 2 Subjects, materials and methods

The study was approved by the Institutional Ethics Committee of Vaszary Kolos Hospital in Esztergom, Hungary. All patients provided written informed consent. A total of 63 patients with diabetes mellitus and carpal tunnel syndrome (DM group; 34 women and 29 men, 73 affected hands), and 146 patients with carpal tunnel syndrome without diabetes (CTS group; 110 women and 36 men, 163 affected hands) were included in the analysis at the Department of Neurology, Vaszary Kolos Hospital, Esztergom, Hungary. The DM group (*n* = 63) comprised 13 patients with type 1 diabetes (DM1; 6 men and 7 women, 15 affected hands) and 50 patients with type 2 diabetes (DM2; 23 men and 27 women, 58 affected hands). The DM group was further subdivided according to presence or absence of electrophysiological signs of polyneuropathy: 43 patients (10 DM1 and 33 DM2; 50 affected hands) with polyneuropathy and 20 patients (4 DM1 and 16 DM2; 25 affected hands) without polyneuropathy.

Inclusion criteria for the CTS group were typical clinical symptoms and signs of carpal tunnel syndrome confirmed by electrophysiological studies. Inclusion criteria for the DM group were typical clinical symptoms and/or electrophysiological abnormalities consistent with carpal tunnel syndrome, together with a diagnosis of diabetes mellitus. The control group consisted of subjects without diabetes or any symptoms suggestive of carpal tunnel syndrome. Exclusion criteria included other endocrine, metabolic, or connective tissue diseases (e.g., thyroid dysfunction, chronic kidney disease, rheumatoid arthritis), as well as pregnancy. Anatomical variations such as a bifid median nerve or premature division of the nerve within the tunnel, prior surgery, or trauma were also exclusion criteria. All patients underwent comprehensive neurological, electrophysiological, and nerve ultrasound examinations.

Fifty age-matched healthy individuals (15 men and 35 women) were enrolled as normal controls. They were divided into two subgroups of 25 participants each, matched to the mean ages of the DM1 and DM2 subgroups (Control 1 and Control 2 groups). None of the control subjects had clinical symptoms or signs suggestive of carpal tunnel syndrome. Nerve conduction studies were not performed in the control subjects. Verbal informed consent was obtained from all control participants.

### 2.1 Nerve conduction studies (NCS)

Nerve conduction studies (NCS) were performed at our institute using a Nicolet Viking Quest EDX System (Carefusion Corporation). The examinations were carried out by the author, who is a physician trained in clinical neurophysiology. In all patients, the examination followed the standard protocol for carpal tunnel syndrome (CTS), which included: median nerve motor and sensory nerve conduction study, segmental antidromic sensory nerve conduction study with palmar and wrist stimulation and ulnar nerve distal motor and sensory nerve conduction study for comparison to confirm carpal tunnel syndrome.

The severity of capal tunnel syndrome was evaluated based on the following parameters: median nerve distal motor latency (DML), motor response (CMAP) amplitude, sensory response (SNAP) amplitude, distal (wrist-finger) sensory conduction velocity and carpal tunnel (palm-wrist) sensory conduction velocity. The severity categories of CTS were defined as by *Csillik* et al.: 1. mild (focal sensory demyelination); 2. moderate (focal sensorimotor demyelination); 3. moderately severe (focal demyelination with sensory axon loss); 4. severe (focal demyelination with sensorimotor axon loss). In our study, we did not distinguish a separate “extremely severe” category; the few cases that would have fallen into this category according to the classification of *Csillik* et al. were evaluated within the “severe” category. Reference values for NCS were collected in our own laboratory with skin temperature controlled (normal limit: DML < 4 ms; distal sensory velocity > 49 m/s; CMAP >4 mV; SNAP: > 10 uV),

In all diabetic patients, motor NCS of the fibular and tibial nerves with F-wave studies, as well as sensory NCS of the superficial radial and sural nerves on the right side, were performed to detect associated polyneuropathy. Electromyography (EMG) was performed at the discretion of the examiner, not on a routine basis. Local reference values for the NCS examinations were used. If abnormalities (decreased nerve conduction velocity or amplitude, increased distal latency or prolonged minimal F-wave latency) were detected in two or more nerves during the electrophysiological examination, the case was classified as diabetic polyneuropathy.

In most control subjects, the right side was examined. The limits of normality were defined as the mean ± 2.5 standard deviations (SD).

### 2.2 Ultrasonography

Ultrasonography was performed by the author, with eight years of experience in nerve ultrasound, using a HITACHI HI VISION Avius UH ultrasound system and an L18–5 MHz linear array transducer. Settings were optimized for nerve imaging, including the use of compound imaging mode. The time interval between ultrasound and electrophysiological testing did not exceed 10 days. Nerve size measurements were performed from palm to mid-forearm in transverse section, and then longitudinal section, on the same side as electrophysiological examination. CSAs were determined altogether at 3 sites: median nerve at the wrist (carpal tunnel inlet, CSA-I), at the palm (carpal tunnel outlet, CSA-O) and on the forearm 12 cm proximal to the wrist. The tunnel inlet was defined as immediately proximal to the proximal edge of the retinaculum, and the tunnel outlet as immediately distal to the distal edge of the retinaculum. Measurements were taken within the hyperechoic external rim of the nerve. Wrist-to-forearm ratio (WFR) and palm-to-forearm ratio (PFR) were also calculated. The same measurements were done in all control subjects on the right side. According to the literature, there is no significant side difference of nerve size *(Zaidman* et al. *2009)*. At each site, three measurements were obtained, and the mean value was used for further analysis to calculate group mean values and standard deviations.

### 2.3 Statistical analysis

Statistical analyses were performed using Statistica for Windows, version 12 (StatSoft, Tulsa, OK, USA). A *p*-value of <0.05 was considered statistically significant. Descriptive statistics were used to summarize demographic data, cross-sectional area (CSA), wrist-to-forearm ratio (WFR), and palm-to-forearm ratio (PFR) values. The Shapiro–Wilk W test was applied to assess the normality of data distribution. Depending on the distribution, comparisons of CSA, WFR, and PFR values between patient and control groups were made using either the unpaired *t*-test (for normally distributed data) or the Mann–Whitney *U* test (for non-normally distributed data). Comparisons between subgroups classified by tunnel syndrome severity and subgroups with or without polyneuropathy were conducted using the Kruskal–Wallis test. Receiver operating characteristic (ROC) curve analysis was used to determine the optimal cut-off values of CSA-I, CSA-O, WFR, and PFR, providing the highest sensitivity and specificity for distinguishing patients from controls. The sensitivity, specificity, positive and negative predictive values, and diagnostic accuracy of CSA-I, CSA-O, WFR, PFR, and their combinations were calculated.

## 3 Results

The mean age in the DM group was 62.2 years (SD: 11.2; range: 24–82), in the CTS group 60.9 years (SD: 14.4; range: 20–85), and in the control group 54.4 years (SD: 16.7; range: 25–83). The age difference between the DM and CTS groups (*p* = 0.21, Kruskal–Wallis test) and between the CTS and control groups (*p* = 0.06, Mann–Whitney *U* test) was not significant. The mean age of the DM group was higher than that of the controls (*p* = 0.002, Mann–Whitney *U* test); however, no difference was found between the DM1/DM2 subgroups and their corresponding control groups (Control 1, Control 2) (*p* = 0.06 and *p* = 0.78, Mann–Whitney *U* test).

Table 1 the median CSA values and WFR/PFR ratios of the patient and control groups, as well as the *p*-values of the statistical comparisons between them. The data were stratified according to the following categories: a) presence of diabetes; b) severity of the carpal tunnel syndrome; c) presence of polyneuropathy; c) ROC-analysis.

**Table 1:** Median values of CSA-I, CSA-O, WFR, PFR and significance levels in the patient and control groups.

	**CSA-I**	**CSA-O**			**Forearm**			**WFR**	**PFR**		
	(mm^2^)	(mm^2^)			(mm^2^)						
	**(IQR)**	**(IQR)**	p*	p**	**(IQR)**	p*	p**	**(IQR)**	**(IQR)**	p*	p**
**CTS**	14.0	13.0	**<0.001**	0.3	5.0	0.1	**0.002**	2.40	2.30	**<0.001**	**0.007**
(*n* = 163)	(11.0–17.0)	(10.0–15.0)			(5.0–6.0)			(2.00–3.00)	(1.83–2.80)		
**DM**	13.0	12.0	**<0.001**		6.0	**<0.001**		2.20	2.20	**<0.001**	
(*n* = 73)	(11.0–16.0)	(10.5–16.0)			(5.0–7.0)			(1.67–2.80)	(1.71–2.67)		
**Control**	9.0	9.0			5.0			1.70	1.70		
(*n* = 50)	(7.5–10.0)	(8.0–10.0)			(5.0–6.0)			(1.50–1.80)	(1.05–2.00)		
											
**DM 1**	13.5	11.0	**<0.05**		5.5	0.63		2.40	2.00	**<0.05**	
(*n* = 15)	(12.0–17.0)	(9.0–17.0)			(5.0–6.0)			(2.00–3.45)	(1.76–3.30)		
**DM 2**	12.0	12.5	**<0.001**		**6.0**	**0.002**		2.1	2.1	**<0.05**	
(*n* = 58)	(10.0–15.3)	(11.0–16.0)			(5.0–7.0)			(1.60–2.65)	(1.70–2.54)		
**Control 1**	9.0	9.0			5.0			1.70	1.70		

CTS: non-diabetic patients with carpal tunnel syndrome; DM: diabetic patients with carpal tunnel syndrome; DM 1: patients with type 1 diabetes; DM 2: patients with type 2 diabetes; Control 1: control group of DM 1; Control 2: control group of DM 2; CSA-I: cross-sectional area at the inlet; CSA-O: cross-sectional area at the outlet; WFR: wrist-to-forearm CSA ratio; PFR: palm-to-forearm CSA ratio; IQR: interquartile range; highlighted in bold: statistically significant difference; p*: CTS/DM v. control (Mann-Whitney *U* test); p**: CTS v. DM (Krustal-Wallis test); CSA values are rounded to the nearest decimal, WFR/PFR, *P* values are rounded to the nearest hundredth.

### 3.1 Presence of diabetes

The CSA-I/CSA-O values of the patient groups were larger and the WFR/PFR ratios were significantly higher than those of the controls Table 1. In patients with diabetes, WFR/PFR ratios were significantly lower (*p* = 0.007, Mann–Whitney *U* test), and forearm CSAs were significantly larger (*p* = 0.002, Mann–Whitney *U* test) compared to CTS patients without diabetes. However, there were no differences in CSA-I/CSA-O values (*p* = 0.3 and *p* = 0.9, Mann–Whitney *U* test) of the patient groups. When analyzed by diabetes type, similar findings were observed in patients with type 2 diabetes. No differences were found between the groups in terms of the disease severity (*p* = 0.4, chi-square test).

### 3.2 Severity of carpal tunnel syndrome

Fig. 3 shows the analysis of the carpal tunnel syndrome severity in patients with and without diabetes. In the CTS group, both the CSAs and the WFR/PFR ratios increased progressively with greater disease severity. In patients with diabetes, WFR/PFR ratios were lower and forearm CSAs were larger in the group with moderately severe carpal tunnel syndrome, compared to CTS subgroup with similar severity. Moreover, the CSA-I/ CSA-O values measured in the moderately severe diabetic subgroup, as well as the WFR/PFR and the forearm CSAs measured in the severe diabetic subgroup, showed small but statistically significant difference compared to the values of the CTS subgroup with similar severity.

**Fig. 3:**
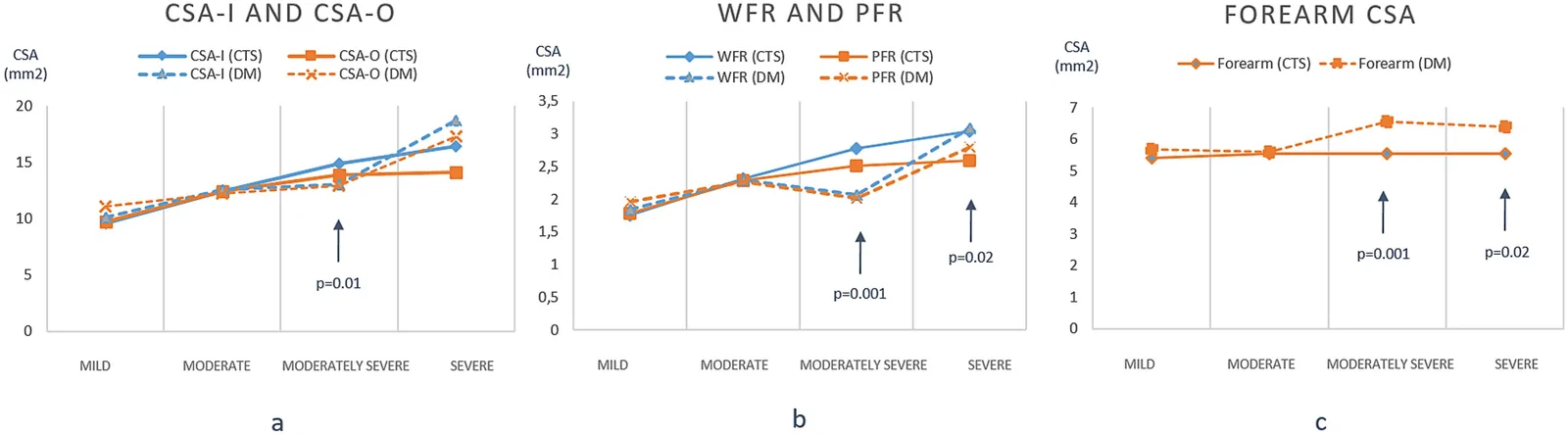
Parameters of the median nerve according to carpal tunnel syndrome severity (median values). In the CTS group, both the CSA values and the WFR/PFR ratios increased progressively with greater disease severity. In the diabetic patients with moderately severe carpal tunnel syndrome, the WFR/PFR was significantly lower and the forearm CSA was larger compared to the CTS subgroup with similar severity. CTS: non- diabetic group; DM: diabetic group; CSA-I: cross-sectional area at tunnel inlet; CSA-O: cross-sectional area at tunnel outlet; WFR: CSA wrist-to-forearm CSA ratio; PFR: palm-to-forearm CSA ratio; arrow: statistically significant difference (Krustal-Wallis test).

### 3.3 Presence of polyneuropathy

Table 2 shows the *p*-values of the statistical comparison between patient subgroups with and without polyneuropathy, within the DM group. The WFR was lower in the DM subgroup with polyneuropathy compared to those without polyneuropathy, although the difference was small (*p* = 0.03, Kruskal–Wallis test). No difference was found between the groups in terms of the disease severity (*p* = 0.3, chi-square test).

**Table 2:** Median values of CSA-I, CSA-O, WFR, PFR and the significance levels of the DM subgroups a**ccording** polyneuropathy.

**Polyneuropathy**	**CSA-I** (mm^2)^	**CSA-O** (mm^2)^	**Forearm** (mm^2^)	**WFR**	**PFR**
	**(IQR)**	**(IQR)**	**(IQR)**	**(IQR)**	**(IQR)**
**Yes**	12.0	12.5	6.0	2.20	2.00
	(10.0–16.0)	(10–16)	(5.0–7.0)	(1.70–2.57)	1.65–2.43)
**No**	14.0	14.0	6.0	2.14	2.36
	(11.0–17.0)	(11.5–20.0)	(5.7–7.0)	(1.80–2.70)	(1.71–3.00)
**P**	0.34	0.05	0.45	**0.03**	0.18

CSA-I: cross-sectional area at the inlet; CSA-O: cross-sectional area at the outlet; WFR: wrist-to-forearm CSA ratio; PFR: palm-to-forearm CSA ratio; IQR: interquartile range; highlighted in bold: statistically significant difference.

### 3.4 ROC-analysis

Tables 3 show the sensitivity, specificity, positive and negative predictive values, and accuracy of CSA and WFR/PFR in each patient group, using optimal cutoff values (CSA-I/CSA-O:10.5 mm2; WFR/PFR:1.85). No differences were found in CSA values between the patient groups; however, in the DM group, the sensitivity of WFR/PFR was 10.4%/10.5% lower, the positive predictive value of WFR/PFR was 13.9%/13.6% lower, and the accuracy of WFR was 8.2% lower compared to the values in the CTS group. When CSA-I/CSA-O values were combined with WFR/PFR, in the case of sensitivity and accuracy, these differences disappeared.

**Table 3:** Sensitivity, specificity, positive/ negative predictive value, accuracy of CSA-I, CSA-O, WFR, PFR in patient groups.

	**CSA-I (mm**^**2**^**)**	**CSA-O (mm**^**2**^**)**	**WFR**	**PFR**	**CSA-I** **+** **WFR**	**CSA-O** **+** **PFR**
**CTS**						
**Sensitivity**	82.2%	71.2%	83.4%	72.0%	97.1%	92.0
	(134/163)	(116/163)	(136/163)	(118/163)	(158/163)	(150/163)
**Specificity**	90.0%	90.0%	76.0%	74.0%	68.4%	66.6%
	(45/50)	(45/50)	(38/50)	(37/50)	(34/50)	(33/50)
**PPV**	96.4%	95.9%	91.9%	90.1%	90.8%	89.8%
	(134/139)	(116/121)	(136/148)	(118/131)	(158/174)	(150/167)
**NPV**	60.8%	48.9%	58.5%	45.1%	87.2%	71.7%
	(45/74)	(45/92)	(38/65)	(37/82)	(34/39)	(33/46)
**Accuracy**	84.0%	75.6%	81.7%	72.8%	71.3	69.1
	(134 + 45/213)	(116 + 45/213)	(136 + 38/213)	(118 + 37/213)	(158 + 34/213)	(150 + 33/213)
**DM**						
**Sensitivity**	76.1%	74.6%	73.0%	61.9%	93.6%	93.5%
	(48/63)	(47/63)	(46/63)	(39/63)	(59/63)	(59/63)
**Specificity**	90.0%	92.0%	74.0%	76.0%	66.6%	69.9%
	(45/50)	(46/50)	(37/50)	(38/50)	(33/50)	(35/50)
**PPV**	90.6%	92.2%	78.0%	76.5%	77.6%	79.7%
	(48/53)	(47/51)	(46/59)	(39/51)	(59/76)	(59/74)
**NPV**	75.0%	74.2%	68.5%	61.3%	89.2%	89.7%
	(45/60)	(46/62)	(37/54)	(38/62)	(33/37)	(35/39)
**Accuracy**	82.3%	82.3%	73.5%	68.1%	69.3	72.3
	(48 + 45/113)	(47 + 46/113)	(46 + 37/113)	(39 + 38/113)	(59 + 33/113)	(59 + 35/113)

CTS: non-diabetic patients with carpal tunnel syndrome; DM: diabetic patients with carpal tunnel syndrome; CSA-I: cross-sectional area at the inlet; CSA-O: cross-sectional area at the outlet; WFR: wrist-to-forearm CSA ratio; PFR: palm-to-forearm CSA ratio; PPV: positive predictive value; NPV: negative predictive value; values are rounded to the nearest decimal.

## 4 Discussion

The aim of this prospective nerve ultrasound study was to compare the neurosonographic parameters of the patients with diabetic and non-diabetic carpal tunnel syndrome, and to examine whether this diagnostic method is equally useful in diabetic carpal tunnel syndrome as in non-diabetic cases.

The literature data *(*Moon et al., 2014*;* Kim et al., 2014*;* Steinkohl et al., 2019*)* regarding nerve size at the wrist in diabetic carpal tunnel syndrome are contradictory, since the study populations are heterogeneous in terms of the carpal tunnel syndrome severity and the diabetes duration. Pooled data *(*Chen et al., 2020*)* did not show significant nerve enlargement in diabetic patients compared to non-diabetic patients, which is consistent with the findings of this study. These results — together with the observation that CSA values were higher in both patient groups than in the control group — suggest that the median nerve CSA can be considered as an accurate diagnostic parameter also in diabetic carpal tunnel syndrome.

However, the WFR/PFR, which represents an additional ultrasonographic feature of the disease, was significantly lower in diabetic than non-diabetic carpal tunnel syndrome. Since this parameter also takes into account the CSA measured at the forearm, it helps reduce inter-individual variability (Hobson-Webb et al., 2008). Moon et al. (2014) demonstrated that WFR/PFR is predictive of carpal tunnel syndrome in diabetic patients as well, whereas Steinkohl et al. (2019) reported that this ratio is significantly lower in diabetic than in non-diabetic CTS, consistent with the findings of the present study.

This aligns with the findings of Lee et al. (2018), who emphasized the need for diabetes-specific ultrasound cut-off values for carpal tunnel syndrome. Furthermore, the present observations also correspond to previous ultrasound studies of upper and lower limb nerves in diabetes *(*Tóth et al., 2023*),* which showed moderate, diffuse nerve enlargement at both compression and non-compression sites. In this context, an increased median nerve forearm CSA reduces the WFR/PFR. Presently, the sensitivity, positive predictive value, and accuracy of WFR/PFR were also significantly lower in diabetic than in non-diabetic patients, suggesting that this parameter may be less reliable in diabetes. At the same time, it was also demonstrated that the combined use of CSA and WFR improve the accuracy at the diagnose of carpal tunnel syndrome in patients with diabetes.

Several methods based on electrodiagnostic findings have been developed to classify the severity of CTS. One of the most widely used is the classification developed by Padua et al. *(*1997*)*, which distinguishes six severity subgroups (negative, mild, moderate, severe, and extreme CTS). Another commonly used classification system was developed by Stevens (1997), according to which CTS is classified as mild, moderate, or severe. Based on additional EMG measurements, Jung et al. (2024) also distinguished an intermediate group alongside the mild, moderate, and severe groups. In our study, we used the classification previously applied by Csillik et al. *(*2016), which appropriately reflects the principle that CTS severity is determined by the degree of axonal loss.

Interestingly, the above-mentioned differences in WFR/PFR were most pronounced in the group with moderately severe carpal tunnel syndrome, which, according to our classification, was characterized by focal demyelination and sensory axonal loss (Fig. 3). The reason why nerve enlargement appears to be more pronounced at this stage remains speculative. It is possible that moderately severe carpal tunnel syndrome is more frequently associated with diabetic polyneuropathy, which may increase forearm CSA and thereby decrease WFR/PFR. This is consistent with the observations of Breiner et al. *(*2017*)* and Tandon et al. *(*2021*)*, who reported a small but statistically significant increase in CSA in diabetic polyneuropathy, particularly in the upper limbs. These findings are also consistent with the results of our previous study, in which we found that, in patients with diabetes, nerve enlargement is observed not only at compression sites but also at non-compression sites **(**Lee et al., 2018).

In addition to the presence of polyneuropathy, several other factors may influence CSA values. Although age differences between patient groups and diabetes subgroups could theoretically affect the results, previous studies have not demonstrated a consistent association between age and nerve size *(*Böhm et al., 2014*)*. In this study, beyond the severity of carpal tunnel syndrome, the type of diabetes also influenced nerve size. The differential effects of diabetes type on nerve morphology have been investigated to a limited extent, as most studies have focused primarily on type 2 diabetes. Consistent with our findings, Breiner et al. (2017) also reported larger CSA values in type 2 diabetes, including at non-compression sites. The difference in nerve size between type 1 and type 2 diabetes may reflect distinct pathophysiological mechanisms underlying diabetic polyneuropathy. A greater tendency toward endoneurial edema has been described in type 2 diabetes, likely related to increased aldose reductase activity within the polyol pathway *(*Singh et al., 2021*).*

To conclude, our prospective study has shown that the median nerve cross-sectional area measured at the inlet and outlet of the carpal tunnel is an independent parameter of diabetes status and serves as an accurate diagnostic marker for carpal tunnel syndrome. The use of the wrist/palm-to-forearm ratio may increase the diagnostic utility of median nerve cross-sectional area measurements; however, in patients with diabetes, it is a less accurate marker compared to non-diabetic individuals, depending on the severity of carpal tunnel syndrome and the presence of concomitant polyneuropathy. However, the combined application of ultrasound parameters enhances diagnostic accuracy, therefore in patients with diabetes, particularly those presenting with moderately severe carpal tunnel syndrome and type 2 diabetes, it is recommended to consider multiple ultrasound parameters. Alternatively, the application of modified reference values tailored to this patient population also could be considered. This approach is less advisable, as a truly reliable diagnostic parameter should remain applicable irrespective of the presence or absence of diabetes.

Limitations of our study include the fact that the physician who performed the ultrasound examination was not „blind” to the electrophysiological results due to limited staffing. In the absence of blinding the correlation between the two methods may be overestimated. Patients with small-fiber neuropathy, which is also characteristic of diabetes, were excluded, as electrophysiological testing primarily detects large-fiber polyneuropathy *(*Ishibashi et al., 2015*).* Furthermore, clinical variables - such as diabetes duration, glycemic control (HbA1c), body mass index, or diabetes treatment - were not included in the analysis, which may theoritecally have a small effect on nerve morphology*.* Another limitation is that only cross-sectional area values were used for morphological analysis, while other ultrasound-based techniques, such as echogenicity assessment, were not performed.

## Declaration of competing interest

The authors declare that they have no known competing financial interests or personal relationships that could have appeared to influence the work reported in this paper.
